# Optimisation of an immunohistochemistry method for the determination of androgen receptor expression levels in circulating tumour cells

**DOI:** 10.1186/1471-2407-14-226

**Published:** 2014-03-28

**Authors:** Jeffrey Cummings, Robert Sloane, Karen Morris, Cong Zhou, Matt Lancashire, David Moore, Tony Elliot, Noel Clarke, Caroline Dive

**Affiliations:** 1 Clinical and Experimental Pharmacology Group, Cancer Research UK Manchester Institute, University of Manchester, Manchester Cancer Research Centre, Manchester M20 4BX, UK; 2 Department of Clinical Oncology, Christie NHS Foundation Trust, Wilmslow Road, Manchester M20 4BX, UK; 3 Urology, Christie NHS Foundation Trust, Wilmslow Road, Manchester M20 4BX, UK

**Keywords:** AZD3514, Immunohistochemistry, Method validation, Incurred sample reanalysis, Cohen’s Kappa, β-Content γ-confidence tolerance intervals

## Abstract

**Background:**

AZD3514 inhibits and down regulates the androgen receptor (AR) and has undergone clinical trials in prostate cancer. To provide proof-of-mechanism (POM) in patients, an immunohistochemistry (IHC) method for determination of AR in circulating tumour cells (CTC) was developed and validated.

**Methods:**

After an assessment of specificity validation focused on intra- and inter-operator reproducibility utilising a novel modification of incurred sample reanalysis (ISR). β-Content γ-confidence tolerance intervals (BCTI) and Cohen’s Kappa (κ) were employed in statistical analysis of results.

**Results:**

In a first set of IHC reproducibility experiments, almost perfect agreement was recorded (κ=0.94) when two different operators scored CTC as overall positive or negative for AR. However, BCTI analysis identified a specific bias in scoring staining intensity, where one operator favoured moderate over strong assignments, whereas the reverse was the case with the second operator. After a period of additional training involving deployment of a panel of standardised images, a second set of validation experiments were conducted. These showed correction of the inter-operator bias by BCTI with κ for scoring intensity increasing from 0.59 to 0.81, indicative of almost perfect agreement.

**Conclusions:**

By application of BCTI to the validation of IHC, operator bias and therefore poor reproducibility can be identified, characterised and corrected to achieve a level of error normally associated with a quantitative biomarker assay, such as an ELISA. The methodological approach described herein can be applied to any generic IHC technique.

## Background

The androgen receptor (AR) axis is a major effector in the development and progression of prostate cancer and an important target in the rational drug design of new anticancer agents [1]. Prior to interaction with ligand (principally 5α-dihydrotestosterone, testosterone and androstenedione) the AR is localised in the cytoplasm bound to heat shock proteins and remains pre-dominantly inactive [2,3]. Upon binding of an androgen the receptor dissociates from heat shock proteins and translocates to the nucleus where it binds to androgen response elements located in the promoter and enhancer regions of target genes, resulting eventually in the formation of an active transcription complex after recruitment of co-regulatory proteins [2,3].

AZD3514 [6-(4-{4-[2-(4-acetylpiperazin-1-yl)ethoxy]phenyl}piperidin-1-yl)-3-(trifluoromethyl)-7,8-dihydro [1,2,4]triazolo[4,3-b]pyridazine] emerged as the preferred clinical candidate from an extensive programme of rational drug design aimed at identifying small molecule inhibitors of the AR [4,5]. The drug has been shown to work by binding to the AR with high affinity, preventing nuclear translocation of the protein and inhibiting ligand-dependent and independent transcriptional activity [4,6]. Unique to AZD3514 as a pharmacological modulator is its ability to down regulate the protein both in vitro and in vivo [6] and its anti-proliferative activity against Dunning R3327H prostate tumours in rats correlated to a reduction in AR levels in tumour tissue [6]. Also, activity of this class of drug is significantly enhanced in animal models by castration. Therefore, AZD3514 entered phase I trials as an oral agent in patients with castration-resistant prostate cancer (CRPC) [7].

During the phase I trial of AZD3514 (EudraCT 2010-020232-19, NCT01162395), detection and enumeration of circulating tumour cells (CTC) pre- and post-administration by the CellSearch System [8] was the major focus of the pharmacodynamic assessment of the drug [7]. Several previous studies have demonstrated that pre-dose CTC numbers around a cut-off value of 5 per 7.5 ml of blood are prognostic of outcome in CRPC (where ≥ 5 predicts for bad prognosis) [9-11]. Thus, CTC were incorporated primarily as a surrogate marker of the anti-tumour activity of AZD3514.

In the present study an immunohistochemistry (IHC) method has been developed for the determination of AR expression in CTC and subject to assay validation for potential application to the clinical evaluation of AZD3514. CTC were harvested from blood samples collected from the Astra Zeneca Sponsored Clinical Study (D1330N00013) by an approach termed Isolation by Size of Epithelial Tumour Cells (ISET), which lends itself more readily to IHC than the CellSearch System [12]. After an initial evaluation of specificity, validation studies focused on reproducibility [13,14], where advanced statistical techniques, as recently applied to the validation of CTC enumeration by CellSearch, were employed in the analysis of data [15].

## Methods

### Collection of patient samples

Whole blood samples (minimum of 10 ml) were collected from 8 different patients entered into the Astra Zeneca sponsored clinical study D1330N00013: A Methodology Study to Assess the Variability of and Effect of Hormone Therapy on (i) Putative Androgen Regulated Gene Expression in Hair Samples and (ii) Circulating Tumour Cell Numbers and Androgen Receptor Expression in Patients with Prostate Cancer. The male subjects were from either Group 1 with localised prostate cancer with no hormonal treatment or Group 2 with locally advanced/metastatic prostate cancer on castration treatment and their characteristics are reported in Table 1.

**Table 1:** Characteristics of subjects entered into the Astra Zeneca sponsored clinical study D1330N00013 whose blood samples where utilised in the present study

**Patient**	**Age (Median)**	**Performance status (Who)**	**Stage**	**Grade (Gleason)**	**Baseline PSA (Median, ng/ml)**
Group I	71	Normal-Restricted Activity	Localised	Intermediate (5–7)	7.0
Group II	74	Normal-Restricted Activity	Locally Advanced/ Metastatic	High (8–10)	23.2

Written informed consent was obtained from all subjects and the studies were ethically approved by the North West 6 Research Ethics Committee (REC) - Greater Manchester South (Northwest Centre for Research Ethics Committees, 3rd Floor - Barlow House, 4 Minshull Street, Manchester M1 3DZ, UK) and the Declaration of Helsinki Principles was followed. The REC reference number for the study was 10/H1013/13. Specimens were obtained by venipuncture into EDTA tubes, stored at 4°C and processed within 4 hr by ISET as described below.

### Isolation by size of epithelial tumour cells

Isolation of CTC from whole blood by the ISET technique was performed according to the manufacturer’s instructions (Rarecells SAS, Paris, France; formerly Metagenex) [12]. Prior to filtration red blood cells were lysed in MetaBuffer (containing 0.8% formaldehyde, Rarecells, 1:10 v/v). Filtration was then conducted through polycarbonate membranes containing 8 μm pores utilising a 10 place vacuum system (Metablock, Rarecells). Thus, each 10 ml sample yielded 10 individual filter spots, which were stored at −20°C prior to staining for the AR by IHC.

### Immunohistochemistry of the androgen receptor in circulating tumour cells

In order to determine the level of AR expression in CTC, ISET filters were analysed by IHC as briefly detailed below. Each individual ISET filter was attached by a paper clip to a glass slide for further handling. Filters were rehydrated in Tris-buffered saline (TBS). All steps were conducted at room temperature unless otherwise stated. Antigen retrieval of samples was conducted in a water bath at 99°C for 40 min in the presence of 250 ml antigen retrieval solution (catalogue number S1699, DAKO, Cambridge, UK). Next, the filters were washed in TBS and incubated with permeabilisation buffer (0.2% triton in TBS) for 10 min. Spots were then placed on a clean side and incubated with peroxidase block (3% hydrogen peroxidase in methanol) for 30 min, after which they were washed in water. Spots were again transferred to a clean slide and incubated overnight with anti-androgen receptor antibody (clone AR441, DAKO at 1:400 in DAKO antibody diluent) in a humidity chamber at 4°C. After incubation with antibody, filters were washed twice in TBS followed by a wash in water. Filters were then placed on a clean slide, Anti-Mouse EnVision + Dual Link System-HRP (DAKO) was added to each filter, which was then incubated for 1 hr after which they were washed twice in TBS. Filters were stained for 10 min with DAKO Liquid DAB + Substrate Chromogen System for 10 min after which they were washed in water. Filters were counter stained with CytoBlue (Innovex Biosciences, Richom, CA, USA) and finally mounted using Faramount (DAKO).

Filters were scanned on the Bioview Allegro™ Plus Scanner (Bioview, Rehovot, Israel) in brightfield mode, covering the entire area of the 0.6 cm diameter spot (scan area was set at 0.7 cm diameter). This scan area was then presented digitally as multiple image frames, from which CTCs could be selected. The brown staining intensity representing the level of AR expression was graded by the analyst as follows: negative, 1+ − weak, 2++ − moderate, 3+++ − strong.

### Characterisation of the specificity of the ISET/immunohistochemistry methodology for the androgen receptor utilising cells lines treated with AZD3514

AZD3514 was received as a kind gift from Astra Zeneca (Oncology iMED, Alderley Park, Macclesfield, UK) and used as received. LNCaP and PC3 human prostate cancer cell lines were obtained from the American Type Culture Collection (ATCC, LGC Standards, Teddington, UK) and cultured according to ATCC recommendations in RPMI medium 1640 containing foetal bovine serum. Prior to drug treatment, LNCaP cells were cultured for 24 hr in phenol-red free RPMI (Life Technologies Ltd, Paisley, UK) with 10% charcoal stripped foetal bovine serum (Life Technologies). The cells were then incubated for 24 hr with 10 μM AZD3514 in 1% DMSO vehicle or vehicle alone as a control. After drug treatment the cells were harvested. Whole blood for spiking with cell lines was donated by healthy human volunteers according to an ethically approved protocol (North West 6 Research Ethics Committee). Blood was spiked with either LNCaP cells prior to incubation with AZD3514 (positive control), LNCaP cells post drug treatment or PC3 cells as a negative control and were then processed by ISET and stained for AR by IHC, as described above.

### Statistical methods

#### Cohen's kappa coefficient (K)

In order to evaluate the degree of inter-operator agreement in the assignment of a staining intensity to each CTC Cohen's kappa coefficient was calculated using GraphPad QuickCalcs (San Diego, CA, USA) based on formula 1 below [16].

(1) $$\kappa =\frac{\mathrm{Pr}\left(a\right)-\mathrm{Pr}\left(e\right)}{1-\mathrm{Pr}\left(e\right)}$$

Here, Pr(a) is the relative observed agreement among operators, and Pr(e) is the hypothetical probability of chance agreement, obtained using the observed data to calculate the probabilities of each observer randomly assigning each category.

#### β-Content γ-confidence tolerance intervals

Agreement in the staining intensity assigned by different operators was also calculated as a measure of incurred sample reanalysis (ISR) utilising β-content γ-confidence tolerance intervals (BCTI). This statistic yields an upper and lower interval where a specified (β) proportion of measurements will lie with a specified (γ) level of confidence and was calculated as previously reported [17]. In our adaptation of this methodology, where normally a single operator assays the same samples twice, data from a pair of operators who assayed the same samples a single time were substituted into the calculations, as described in full detail recently [15]. Calculation of BCTI was performed utilising MATLAB (Version R2009a, MathWorks, Natick, MA, USA) at β = 67% and 95% [18]. A plot of BCTI (y-axis) against IHC score (x-axis) represents a modified form of the ‘accuracy profile’.

## Results

**Figure 1:**
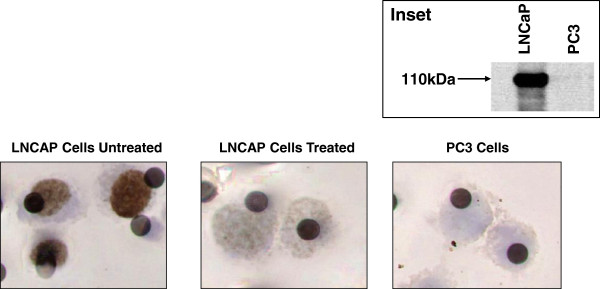
**Characterisation of the specificity of the ISET/immunohistochemistry methodology for the androgen receptor utilising cells lines as controls.** Specificity was investigated by employing human prostate cancer cell lines of known AR status, together with treatment of cells with AZD3514 in order to modulate the levels of the protein, to spike volunteer blood samples prior to processing by ISET and analysis by IHC. Untreated LNCaP cells were the positive control and demonstrated strong brown nuclear staining for AR. PC3 cells were the negative control and displayed a complete absence of brown staining. LNCaP cells pre-treated for 24 hours with 10 μM AZD3514 prior to spiking into blood demonstrated a marked reduction in the level of nuclear staining, compared to untreated LNCaP cells. Inset: Western blot analysis of LNCaP and PC3 cells for androgen receptor protein expression. Loading was normalised by the addition of 20 μg of protein to each lane and the blot was run according the standard western blot protocols, utilising anti-androgen receptor antibody clone AR441 as the primary antibody and enhanced chemiluminescence detection for visualisation of bands.

Specificity of the ISET/IHC technique for the AR in CTC was investigated employing, as controls, human prostate cancer cell lines of known AR status, together with treatment of cells with AZD3514 in order to modulate the levels of the protein. The positive control AR expressing cell line was LNCaP while the negative control was PC3. AR status was confirmed in these cell lines by Western blot analysis utilising the same antibody (AR441) as that employed in the IHC method through the presence of a band at 110 kDa in LNCaP and the absence of a band in PC3 (Inset to Figure 1), in keeping with previously published studies [19]. Figure 1 illustrates the results obtained from a typical ISET/IHC experiment, where blood from healthy volunteers was spiked with either untreated LNCaP cells, PC3 cells or LNCaP cells incubated with AZD3514 at a dose shown to reduce AR protein expression in this cell line [6]. Untreated LNCaP cells - the positive control - demonstrated strong nuclear brown staining for (translocated) AR, whereas the PC3 cells - the negative control - displayed a complete absence of brown staining. The almost complete absence of cytoplasmic staining in LNCaP cells may be due to constitutive autocrine stimulation of the AR signaling pathway [20]. In addition, in LNCaP cells pre-treated with AZD3514 prior to spiking into blood there was a marked reduction in the level of nuclear staining. These data indicate that the ISET/IHC method described herein can distinguish between AR positive and AR negative cancer cells in the blood of human subjects and is also sufficiently sensitive to detect a drug induced (pharmacodynamic) knockdown in protein levels.

The major focus of the present study was to characterise between-operator and within-operator variability, employing multiple blood samples collected from either different patients or at different time points in the same patient. The number of CTCs captured in the different ISET filter spots ranged from 0 to 30 per ml of blood, within the range of CTC previously reported in prostate cancer patients [21].

In the first validation experiment 4 spots from 8 different patient blood samples were stained by IHC for AR expression and presented blindly to two different operators to both enumerate the CTC and score the staining intensity of each cell. The degree of inter-operator agreement was assessed both as a percentage and as κ and is presented in Table 2. Although Cohen's kappa coefficient is a statistical measure of inter-operator agreement for qualitative (categorical) items the κ statistic is not a test of significance. Nonetheless, it is a robust measure since it takes into account random agreement occurring by chance. Guidelines have been published to aid in the interpretation of κ [22], and these have been previously applied to the enumeration of CTC by CellSearch [23]. Significantly, almost perfect agreement was observed when the two different operators scored CTC as overall positive or negative for AR, with a κ value of 0.94. However, when the scores produced by different operators for staining intensity were analysed there was a large reduction in κ from 0.94 to 0.59, indicating a significant degree of disagreement.

**Table 2:** Degree of inter-operator agreement in scoring of CTC for expression of the androgen receptor by immunohistochemistry

	**1**^**st **^**Run**		**2**^**nd **^**Run**	
Evaluation	Kappa^1^	% Agreement	Kappa	% Agreement
Positive v Negative^2^	0.94	97.2	0.98	98.9
Staining intensity^3^	0.59	75.0	0.81	87.9

^1^Kappa values where calculated as described in Methods where the agreement between operators is defined as follows: < 0 none, 0–0.20 slight, 0.21–0.40 fair, 0.41–0.60 moderate, 0.61–0.80 substantial, and 0.81–1 almost perfect [22].

^2^This table entry refers to an overall assessment of the level of positive versus negative staining regardless of the staining intensity.

^3^Staining intensity was graded into 4 categories as negative, weak (1 +), moderate (2 ++) or strong (3 +++).

**Figure 2:**
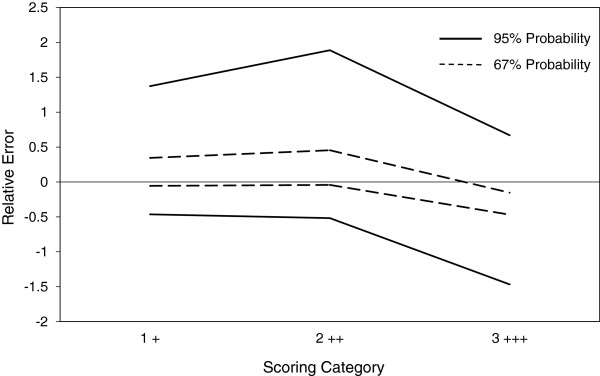
**Characterisation of inter-operator variability in AR staining intensity by IHC in CTC harvested from patient samples by ISET.** ISET membrane spots obtained after filtration of a number of different patient blood samples were stained by IHC for AR expression and presented blindly to two different analysts to both enumerate the CTC and score the staining intensity. Results were then analysed by a modification of ISR where the staining intensities obtained by each operator were substituted into the calculations. β-Content γ-confidence tolerance intervals (±) were calculated at β = 95% and 67% and the resulting accuracy profiles plotted. These revealed a systematic bias characterised by one operator favouring a score of 2 ++ over another favouring 3 +++.

β-Content γ-confidence tolerance intervals (BCTI) reports on ISR both in the form of precision/imprecision and trueness/bias. Figure 2 illustrates the accuracy profiles at both 67% and 95% probability for the staining intensity assignments of two operators. These demonstrate a relatively large degree of imprecision, as might be expected with categorical data. However, they also highlight a significant bias, where operator 1 favoured a score of 2++ (moderate staining intensity) over 3+++ (strong), whereas operator 2 favoured 3+++ over 2++. Such a bias would not be evident by κ alone.

**Figure 3:**
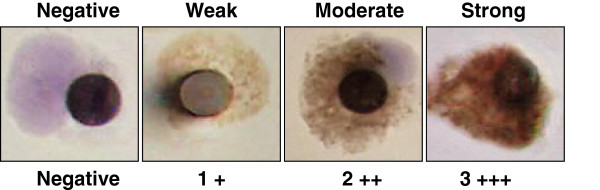
**Typical example of the training set of images used to standardise AR receptor staining intensity in CTC isolated by ISET.** After identifying significant operator bias in assigning staining intensities (see Figure 2), a standard gallery of 20 different images was produced as an aid to staff training. The figure contains a typical set of images from the gallery each containing a single patient derived CTC isolated on an ISET membrane and stained for AR expression by IHC to illustrate the different levels of staining intensity observed and the scoring system utilised in the present study.

**Figure 4:**
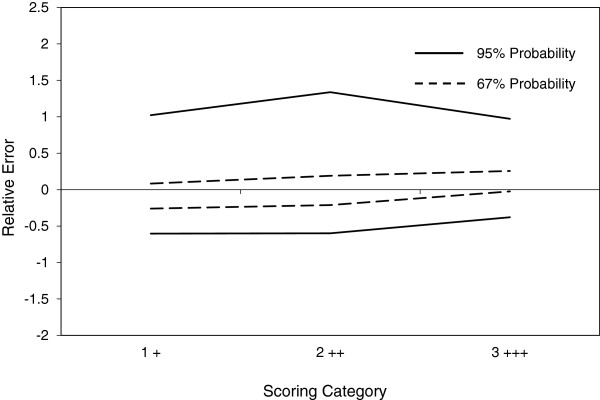
**Further characterisation of inter-operator variability in AR staining intensity by IHC in CTC harvested from patient samples by ISET.** After additional training utilising the standard gallery of images (see Figure 3), the same two analysts were invited to blindly score AR staining intensity in CTC harvested from patient blood samples by ISET. Again, results were analysed by a modification of ISR, β-content γ-confidence tolerance intervals (±) were calculated at β = 95% and 67% and the resulting accuracy profiles constructed. In this case, the bias observed in the first validation experiment (see Figure 2) appeared to be effectively eliminated.

After the first validation experiment a programme of staff training was embarked upon. A gallery of 20 IHC images of CTC harvested by ISET from a number of different patients was constructed in order to facilitate a supervised training workshop. Here analysts were presented with 5 different sets of images where each set included an example of a patient CTC expressing AR at weak, moderate, strong and negative staining levels. One such set of 4 graded images taken from the gallery is illustrated in Figure 3. In the second validation experiment, the bias between operators observed in experiment 1 was completely eliminated (see Figure 4), and the value of κ for inter-operator agreement increased from 0.59 to 0.81 (Table 1), the latter being in the category of almost perfect agreement [22].

**Figure 5:**
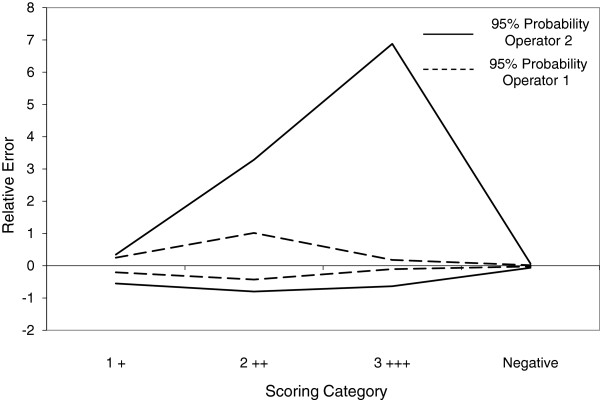
**Incurred sample reanalysis in AR staining intensity determined by β-content γ-confidence tolerance intervals.** Due to the fact that the set of samples analysed blindly by two different operators in the first (Figure 2) and second (Figure 4) validation experiments were identical, this also allowed a conventional analysis of results by ISR utilising β-content γ-confidence tolerance intervals (±) at β = 95%. The resulting accuracy profiles clearly demonstrated that the training programme impacted almost exclusively on operator 2, correcting the between-operator bias in the process (Figure 2). They also highlight that the degree of ISR achievable in this analysis by operator 1 approached 30%, which is the accepted benchmark for total error for a typical quantitative biomarker assay.

Since the samples proffered to the analysts in the second validation experiment were identical to those proffered in the first, this allowed for a conventional assessment of ISR [17] (see Figure 5). These data highlight the effect of the training programme, where analyst 1 showed a greater difference in scoring objects as 2++ between experiments , while analyst 2, as anticipated, showed a greater difference in scoring objects in the 3+++ category. It is also clear that the major effect of the training programme was more manifest on operator 2, while the degree of ISR achieved by operator 1 approached 30%.

## Discussion

Fit-for-purpose biomarker method validation defines 5 categories of assay based on readout ranging from absolute quantitation to a nominal positive/negative result [13,14,24,25]. Along this spectrum, IHC in ocular microscopy mode is identified as an ordinal qualitative assay that yields categorical data presented as discrete scoring scales. Many of the performance characteristics normally associated with bioanalytical methods are not relevant to IHC, for example accuracy [26,27]. However, the main parameter of relevance to a qualitative assay is reproducibility: defined by the ICH as the “precision of repeated measurements between (operators and) laboratories” [14]. Or put more simply, “the property of receiving consistent results from following a specific procedure” [26]. Although there are many technical variables that could impact on the reproducibility of an IHC method, such as processing and embedding tissue and selection of section thickness, [27] the major source of error is recognised as that which is introduced by the reader [28,29].

In the present paper, method validation was performed on an IHC method for the determination of AR in CTC. While the focus was obviously reproducibility in addition to an assessment of specificity, the issue of inter-operator variability was addressed in a novel manner, through a modification of ISR [30,31]. Thus, in addition to conducting ISR in the conventional format (where typically a single operator analyses the same set of samples twice), in our modification two different operators each analysed the same set of samples once and the results were then subjected to statistical analysis by BCTI. We have previously demonstrated that in the case of CTC enumeration by CellSearch this modified approach to ISR was exquisitely sensitive in differentiating between both systematic (bias) and random (imprecision) errors [15]. Our results confirm that even in the relatively less complex scenario of a single CTC sitting on a filter, there is still considerable between-operator variability in the assignment of a scoring intensity. However, since BCTI identifies the nature of the inter-operator error, it also allowed for its correction through additional staff training. In addition, by adoption of this approach it has been demonstrated that a highly trained analyst is capable of achieving scores in the repeat analysis of samples that varied by 30%, which is within the accepted benchmark for total error of a typical quantitative biomarker/pharmacodynamic assay such as an ELISA [14,25,32].

As a categorical assay, IHC has a dynamic range restricted to a limited number (normally 3 to 4) of band widths of staining intensity. Nonetheless, in preclinical studies with AZD3514, and utilising the same scoring structure as the present method (0+, 1+, 2+ and 3+), IHC was able to demonstrate a dose dependent reduction on AR in tumour tissue, at doses of drug that produced only a modest inhibition of tumour growth [6]. In the same report a comparative evaluation was conducted between ocular microscopy and image analysis using the Aperio system (ePathology Solutions, Oxford, UK). Reassuringly, both approaches reported a similar level of knockdown in AR. Among current IHC techniques approved by the FDA as diagnostic, prognostic or predicative biomarkers, no claims are made that image analysis is any more accurate than visual assessment by a trained pathologist [27].

The ISET technique is an example of a tumour-marker-independent technology based on filtration through 8 μm pores, unlike the FDA cleared CellSearch System which relies critically on the presence of epithelial markers (EpCam) and antibody directed capture [21,33]. However, the ISET technique may have an advantage over CellSearch since it is now believed that a significant proportion of malignant CTCs lose their “epithelial markers” in preference to mesenchymal antigens, in a process known as epithelial to mesenchymal transition [34-36]. As a consequence the ISET technique invariably harvests larger populations of CTC from patient blood than the CellSearch system [37]. Nonetheless, while the ISET technique is claimed to effectively remove the majority of hematologic cells - red blood cells by lysis and peripheral blood leukocytes by filtration [12] - rare hematologic cells (megakaryocytes or large monocytes) or mesenchymal (endothelial) cells may be difficult to distinguish from epithelial tumour cells by cytopathologic or immunochemical analysis [38]. Indeed, it has been demonstrated in a number of different disease types, that ISET harvests cells of a non-malignant morphology from a small but significant group of subjects that could potentially result in a false-positive diagnosis [38]. These results advise caution when relying on a single technique to isolate CTC from patients.

## Conclusions

A novel procedure is presented for the fit-for-purpose evaluation of the reproducibility of an IHC method for the determination of AR receptor expression levels in CTC isolated from patients as a biomarker assay utilising a modification of ISR and BCTI for statistical analysis of results. The procedure was employed to identify and correct inter-operator bias in the assignment of a scoring intensity and poor reproducibility, resulting potentially in a reduction of measurement error to a level normally associated with a quantitative biomarker assay, such as an ELISA. The methodological approach could be applied theoretically to any generic IHC method.

## Abbreviations

AR: Androgen receptor; IHC: Immunohistochemistry; CTC: Circulating tumour cells; BCTI: β-content γ-confidence tolerance intervals; K: Cohen’s Kappa; CRPC: Castration-resistant prostate cancer; POM: Proof-of-mechanism; ISET: Isolation by size of epithelial tumour cells; ISR: Incurred sample reanalysis; REC: Research ethics committee.

## Competing interests

The authors declare that they have no competing interests.

## Authors’ contributions

JC, KM, CZ, RS and CD were the main authors of the manuscript. CZ developed and validated all programming code utilised in statistical analysis. JC conducted the statistical analysis and interpretation of data. RS and ML performed all the laboratory analysis of samples. NC and TE collected blood samples and clinical data from patients. All authors have read and approved the final version of the manuscript.

## Pre-publication history

The pre-publication history for this paper can be accessed here:

http://www.biomedcentral.com/1471-2407/14/226/prepub
